# A randomized multicenter clinical trial to evaluate the efficacy of melatonin in the prophylaxis of SARS-CoV-2 infection in high-risk contacts (MeCOVID Trial): A structured summary of a study protocol for a randomised controlled trial

**DOI:** 10.1186/s13063-020-04436-6

**Published:** 2020-06-03

**Authors:** Irene García García, Miguel Rodriguez-Rubio, Amelia Rodríguez Mariblanca, Lucía Martínez de Soto, Lucía Díaz García, Jaime Monserrat Villatoro, Javier Queiruga Parada, Enrique Seco Meseguer, María J. Rosales, Juan González, José R. Arribas, Antonio J. Carcas, Pedro de la Oliva, Alberto M. Borobia

**Affiliations:** 1 Clinical Pharmacology Department, Clinical Trial Unit, La Paz University Hospital – IdiPAZ, Paseo de la Castellana, 261, 28046 Madrid, Spain; 2 School of Medicine, Universidad Autónoma de Madrid, Madrid, Spain; 3 Pediatric Intensive Care Department, La Paz University Hospital – IdiPAZ, Paseo de la Castellana, 261, 28046 Madrid, Spain; 4 Clinical Trial Unit, La Paz University Hospital – IdiPAZ, Madrid, Spain; 5 Internal Medicine Department, Infectious Diseases Unit, La Paz University Hospital – IdiPAZ, Madrid, Spain

**Keywords:** COVID-19, Randomised controlled trial, protocol, melatonin, prophylaxis, healthcare workers

## Abstract

**Objectives:**

Primary objective: to evaluate the efficacy of melatonin as a prophylactic treatment on prevention of symptomatic SARS-CoV-2 infection among healthcare workers at high risk of SARS-CoV-2 exposure.

Secondary objectives:

- To evaluate the efficacy of melatonin as a prophylactic treatment on prevention of asymptomatic SARS-CoV-2 infection.
- To evaluate the efficacy of melatonin to prevent the development of severe COVID-19 in the participants enrolled in this study who develop SARS-CoV-2 infection along the trial.
- To evaluate the duration of COVID-19 symptoms in participants receiving melatonin before the infection.
- To evaluate seroconversion timing post-symptom onset.

Exploratory objectives**:**

- To compare severity of COVID-19 between men and women.
- To evaluate the influence of sleep and diet on prevention from SARS-CoV-2 infection.
- To evaluate the effect of melatonin on the incidence and characteristics of lymphopenia and increase of inflammatory cytokines related to COVID-19.

**Trial design:**

This is a two-arm parallel randomised double-blind controlled trial to evaluate the efficacy of melatonin versus placebo in the prophylaxis of coronavirus disease 2019 among healthcare workers.

**Participants:**

Inclusion Criteria:

- Male or female participants ≥ 18 and ≤ 80 years of age.
- Healthcare workers from the public and private Spanish hospital network at risk of SARS-CoV 2 infection.
- Not having a previous COVID19 diagnosis.
- Understanding the purpose of the trial and not having taken any pre-exposure prophylaxis (PrEP) including HIV PrEP from March 1^st^ 2020 until study enrolment.
- Having a negative SARS-CoV 2 reverse-transcription PCR (RT-PCR) result or a negative serologic rapid test (IgM/IgG) result before randomization.
- Premenopausal women must have a negative urinary pregnancy test in the 7 days before starting the trial treatment.
- Premenopausal women and males with premenopausal couples must commit to using a high efficiency anticonceptive method.

Exclusion Criteria:

- HIV infection.
- Active hepatitis B infection.
- Renal failure (CrCl < 60 mL/min/1.73 m2) or need for hemodialysis.
- Osteoporosis.
- Myasthenia gravis.
- Pre-existent maculopathy.
- Retinitis pigmentosa.
- Bradycardia (less than 50 bpm).
- Weight less than 40 Kg.
- Participant with any immunosuppressive condition or hematological disease.
- Treatment with drugs that may prolong QT in the last month before randomization for more than 7 days including: azithromycin, chlorpromazine, cisapride, clarithromycin, domperidone, droperidol, erythromycin, halofantrine, haloperidol, lumefantrine, mefloquine, methadone, pentamidine, procainamide, quinidine, quinine, sotalol, sparfloxacin, thioridazine, amiodarone.
- Hereditary intolerance to galactose, Lapp lactase deficiency or glucose or galactose malabsorption.
- Treatment with fluvoxamine.
- Treatment with benzodiazepines or benzodiazepine analogues such as zolpidem, zopiclone or zaleplon.
- Pregnancy.
- Breastfeeding.
- History of potentially immune derived diseases such as: lupus, Crohn's disease, ulcerative colitis, vasculitis or rheumatoid arthritis.
- Insulin-dependent diabetes mellitus.
- Known history of hypersensitivity to the study drug or any of its components.
- Patients that should not be included in the study at the judgment of the research team.

Participants will be recruited from the following eight hospitals in Madrid, Spain: Hospital Universitario La Paz, Hospital Ramón y Cajal, Hospital Infanta Sofía, Hospital 12 de Octubre, Hospital Clínico San Carlos, Hospital Central de la defensa Gómez Ulla,Hospital de La Princesa and Hospital Infanta Leonor.

**Intervention and comparator:**

Experimental: Melatonin (Circadin®, Exeltis Healthcare, Spain): 2 mg of melatonin orally before bedtime for 12 weeks.

Comparator**:** Identical looking placebo (Laboratorios Liconsa, Spain) orally before bedtime for 12 weeks.

**Main outcomes:**

Number of SARS-CoV-2 (COVID-19) symptomatic infections confirmed by polymerase chain reaction (PCR) test or serologic test or according to each centre diagnosis protocol. Primary outcome will be measured until the end of treatment for each participant (until the date of the last dose taken by each patient).

**Randomisation:**

Patients who meet all inclusion and no exclusion criteria will be randomised, stratified by centres, sex and age (<50 and ≥ 50 years old). The randomisation sequence was created using SAS version 9.4 statistical software (procedure ‘PROC PLAN’) with a 1:1 allocation. No randomisation seed was specified. The randomisation seed was generated taking the hour of the computer where the program was executed. Randomization will be done centrally through the electronic system RedCAP® in order to conceal the sequence until interventions are assigned

**Blinding (masking):**

Participants, caregivers, and those assessing the outcomes are blinded to group assignment.

**Numbers to be randomised (sample size):**

A total of 450 participants are planned to be enrolled in this clinical trial, 225 in the experimental arm and 225 in the placebo arm.

**Trial Status:**

Protocol version 3.0, 17th of April 2020. Recruitment ongoing.

First participant was recruited on the 21st of April 2020. The final participant is anticipated to be recruited on the 31st of May 2020.

As of May 18th, 2020, a total of 312 participants have been enrolled (154 at Hospital La Paz, 85 at Hospital Infanta Sofía and 73 at Hospital 12 de Octubre).

**Trial registration:**

EU Clinical Trials Register: 2020-001530-35; Date of trial registration: 13th of April 2020; https://www.clinicaltrialsregister.eu/ctr-search/trial/2020-001530-35/ES

**Full protocol:**

The full protocol is attached as an additional file, accessible from the Trials website (Additional file 1). In the interest in expediting dissemination of this material, the familiar formatting has been eliminated; this Letter serves as a summary of the key elements of the full protocol.

## Supplementary information

**Additional file 1.** Protocolo de ensayo clínico.

## Data Availability

Not applicable.

