# Supplementary material for: A randomized multicenter clinical trial to evaluate the efficacy of melatonin in the prophylaxis of SARS-CoV-2 infection in high-risk contacts (MeCOVID Trial): A structured summary of a study protocol for a randomised controlled trial
Source: Trials. 2020 Jun 3;21:466. doi: 10.1186/s13063-020-04436-6 (PMC7267766; doi:10.1186/s13063-020-04436-6)
Supplement: Supplementary file 1 — Additional file 1. Protocolo de ensayo clínico. [file 13063_2020_4436_MOESM1_ESM.pdf]

PROTOCOLO DE ENSAYO CLÍNICO

Ensayo clínico multicéntrico aleatorizado para evaluar la eficacia de melatonina en la profilaxis de la infección por SARS-CoV-2 en contactos de alto riesgo (Ensayo MeCOVID – COVID-19)

Versión protocolo: 3.0, 16 de Abril de 2020

Promotor: Alberto M. Borobia Pérez **Código del Protocolo:** MeCOVID
EudraCT: 2020-001530-35-35

CONFIDENCIAL

La información y los datos incluidos en este protocolo es propiedad del Promotor. Ninguna persona está autorizada a hacer pública dicha información sin el permiso por escrito del Promotor. Este material podrá ser divulgado y utilizado por su equipo y asociados, en la medida en que pueda ser necesario para el desarrollo del estudio clínico.

RESUMEN 4
INFORMACIÓN GENERAL 8
Investigador coordinador 8
Comite Cientifico 8
Centros participantes 8
Identificación del monitor. 9
Comité de Ética evaluador 9
Duración prevista del ensayo 9
ANTECEDENTES Y JUSTIFICACIÓN 10
INTRODUCCIÓN 10
JUSTIFICACIÓN DEL USO DE MELATONINA EN PROFILAXIS 12
PLANTEAMIENTO DEL ESTUDIO 15
Diseño de ensayo. 15
Hipótesis 15
Objetivos. 15
SELECCIÓN DE PARTICIPANTES. 16
Criterios de inclusión: 16
Criterios de exclusión: 16
Subestudio InmunoCOVID 17
Tamaño de la muestra. 17
Grupos de tratamiento. 18
Criterios de retirada y abandono. 19
DESARROLLO DEL ESTUDIO Y PROCEDIMIENTOS. 20
Evaluaciones y procedimientos del estudio. 20
Calendario de evaluaciones 21
Visitas y procedimientos. 22
Seguimiento. 27
Variable Principal. 28
Variable secundaria: 28
SEGURIDAD DEL ESTUDIO 30
Información de seguridad de deferencia 30
Definiciones 30
ACONTECIMIENTO ADVERSO (AA)) 30
Acontecimiento Adverso Grave (AAG) 31
Reacción Adversa (RA) 31
Evaluación de la causalidad. 32
Evaluación de la intensidad. 32
Embarazos 33
Procedimiento para la notificación de los acontecimientos adversos graves 33
Procedimiento para la notificación de los acontecimientos adversos graves e inesperados (RAGI) 34

ASPECTOS ETICOS 36
Normas generales y particulares para los investigadores. 36
Dispositivos de seguridad y confidencialidad. 36
CONSIDERACIONES PRÁCTICAS 36
Responsabilidades de los participantes en el estudio 37
Monitorización, auditoría e inspección 38
Documentación del estudio 38
Manejo y archivo de datos 38
Condiciones de publicación 39
Procedimiento para las modificaciones del protocolo 39
Comité ético de investigación con medicamentos (CEIm) 40
BLBIBLIOGRAFÍA 41

RESUMEN

Promotor: Alberto M. Borobia Pérez
Código del Protocolo: MeCOVID
EudraCT: 2020-001530-35

Investigador coordinador

Dr. Alberto M. Borobia
Hospital Universitario La Paz.
Servicio de Farmacología Clínica
Paseo de la Castellana, 261 CP 28046 Madrid
Teléfono +34912071466 /Fax +34912071466

Comité Científico

Servicio de Farmacología Clínica
Dr. Antonio J. Carcas
Dra. Irene García García
Dra. Amelia Rodríguez Mariblanca
Dra. Lucía Martínez de Soto
Dra. María José Rosales
Servicio de Cuidados Intensivos Pediátricos
Dr. Pedro de la Oliva Senovilla
Dr. Miguel Rodríguez Rubio
Servicio de Medicina Interna. Enfermedades Infecciosas
Dr. José Ramón Arribas
Dr. Juan González

Centros participantes

Centro	Investigador Principal
**Hospital Universitario La Paz**	Juan González
**Hospital Ramón y Cajal**	Begoña Monge
**Hospital Infanta Sofía**	Pilar Rúiz
**Hospital 12 de Octubre**	Antonio Lalueza
**Hospital Clínico San Carlos**	Vicente Estrada
**Hospital Central de la defensa Gómez Ulla**	Javier Membrillo
**Hospital de La Princesa**	Jesus Sanz
**Hospital Infanta Leonor**	Guillermo Cuevas

Podrán adherirse más hospitales nacionales, previa solicitud de autorizacion al CEIm y AEMPs.

Fase del ensayo clínico

Ensayo clínico multicéntrico , aleatorizado doble ciego y controlado con placebo.

Comité de Ética evaluador

El estudio será evaluado por el Comité de Ética de la Investigación con medicamentos (CEIm) del Hospital Universitario La Paz acreditado por el Ministerio de Sanidad y por las autoridades sanitarias de la Comunidad Autónoma de Madrid.

Duración prevista del ensayo

La duración de estudio será la de la pandemia por COVID19 en las áreas geográficas de los centros participantes. El tiempo de duración del tratamiento será de 12 semanas. El seguimiento de los participantes se realizará hasta 4 semanas después de la última toma.

Resumen de la justificación

Existe la necesidad urgente de evaluar mediante ensayos clínicos las potenciales intervenciones que puedan prevenir la infección por SARS-CoV-2 en personal hospitalario con alto riesgo de contagio. La melatonina se postula como un potencial candidato por su actividad antiinflamatoria, antioxidante y protectora, en infecciones bacterianas y virales, tratándose además de un producto barato, accesible y con escasos efectos secundarios descritos.

Enfermedad de estudio

Enfermedad por nuevo coronavirus SARS-CoV-2, COVID-19.

Diseño

El diseño del estudio es un ensayo clínico multicéntrico, aleatorizado doble ciego y controlado con placebo que tiene por objetivo principal evaluar la eficacia de melatonina en la prevención de la infección por SARS-CoV-2 en personal hospitalario con contactos de alto riesgo.

Tratamiento en evaluación.

Se hará una aleatorización estratificada por sexo, edad (<50 y > o =50 años) y centro a través de la aplicación RedCAP, a uno de los dos siguientes grupos de tratamiento (1:1):

- **Grupo 1:** Melatonina 2 mg diarios, antes de acostarse, durante 12 semanas.
- **Grupo 2:** Placebo de aspecto idéntico 2 mg diarios, antes de acostarse, durante 12 semanas.

Los participantes con positivización de la PCR y/o serología y/o test de diagnóstico rápido para SARS-CoV-2 a lo largo del estudio pasarán a manejo según práctica clínica habitual, pero se realizará seguimiento hasta resolución de la enfermedad o fallecimiento.

Población estudio

Se seleccionarán hombres y mujeres adultos que formen parte del personal hospitalario que directa o indirectamente estén expuestos a la infección por coronavirus en el ejercicio profesional.

Para que los resultados sean comparables a los resultados del Ensayo clínico para prevención de la Infección por coronavirus en sanitarios que está poniendo en marcha el Ministerio de Sanidad, (Ensayo clínico EPICOS) asumimos sus mismos criterios de inclusión y exclusión.

Criterios de inclusión:

- Hombre y mujeres con edad entre 18 y 70 años.
- Forman parte del personal hospitalario de hospitales públicos o privados de la red hospitalaria española en una zona con riesgo de transmisión de SARS-CoV-2.
- No haber sido diagnosticado previamente de infección por SARS-CoV-2 (COVID-19).
- Entender el propósito del estudio y NO haber tomado ninguna medicación como PrEP frente a SARS-CoV-2 desde el 1 de marzo de 2020 hasta la entrada en el ensayo (también incluye PrEP para el VIH).
- Tener una PCR o test de diagnóstico rápido IgM/IgG para SARS-CoV-2 negativo a la entrada.
- Prueba de embarazo en orina negativa realizada en los 7 días anteriores al comienzo del tratamiento en estudio en mujeres pre-menopáusicas o < 2 años después de la menopausia.
- Las mujeres en edad fértil y los varones con pareja en edad fértil deben comprometerse a utilizar un método anticonceptivo de gran eficacia (como esterilización quirúrgica, método de doble barrera, anticonceptivos orales o implantes hormonales contraceptivos) y a continuar utilizándolos hasta el día de la última dosis de tratamiento.

Criterios de exclusión:

- Infección por el VIH.
- Infección activa por virus de la hepatitis B.
- Insuficiencia renal (aclaramiento de creatinina < 60 ml/min/1.72 m^2) y pacientes en hemodiálisis.
- Osteoporosis.
- Miastenia gravis.

- Maculopatía preexistente del ojo.
- Retinitis pigmentosa.
- Bradicardia < 50 latidos/minuto.
- Peso < 40 Kg.
- Participantes con enfermedad inmunosupresora o hematológica.
- Tratamiento en el último mes antes de la aleatorización y durante más de 7 días, con fármacos que pueden prolongar el intervalo QT incluidos: azitromicina, clorpromazina, cisaprida, claritromicina, domperidona, droperidol, eritromicina, halofantrina, haloperidol, lumefantrina, mefloquina, metadona, pentamidina, procaina quinidina, quinina, sotalol, esparfloxacina, tioridazina, amiodarona.
- Los participantes que presenten intolerancia hereditaria a la galactosa, de insuficiencia de lactasa de Lapp o malabsorción de glucosa o galactosa.
- Tratamiento con fluvoxamina.
- Tratamiento con benzodiacepinas o hipnóticos no benzodiacepínicos, tales como el zaleplón, el zolpidem y la zopiclona.
- Embarazo o planificación de quedarse embarazada durante el transcurso del estudio. Lactancia
- Participantes con historia de enfermedades potencialmente mediadas por el sistema inmune o enfermedades inflamatorias: lupus eritematoso sistémico, Crohn, colitis ulcerosa, vasculitis, artritis reumatoide.
- Hipersensibilidad al principio activo o a alguno de los excipientes.
- Participantes que por cualquier motivo no deberían ser incluidos en el estudio según evaluación del equipo investigador.

Tamaño muestral

Para un poder estadístico de al menos el 90% y un error alfa del 5%, se necesitan aproximadamente **450** sujetos para demostrar nuestra hipótesis.

Seguimiento

Se realizará seguimiento de los participantes hasta 4 semanas tras la última toma del medicamento. En los participantes con positivización de la PCR y/o serología para SARS-CoV-2 se realizará seguimiento hasta resolución de la enfermedad o fallecimiento.

Objetivos

El objetivo principal es evaluar la eficacia de melatonina en la prevención de la infección por SARS-CoV-2 en personal hospitalario con contactos de alto riesgo.

Se evaluará además la eficacia de melatonina en la prevención del desarrollo de COVID-19 grave en participantes que adquieran la infección durante el desarrollo del estudio.

INFORMACIÓN GENERAL

Promotor: Alberto M. Borobia Pérez
Código del Protocolo: MeCOVID
EudraCT: 2020-001530-35

Investigador coordinador

Dr. Alberto M. Borobia
Hospital Universitario La Paz.
Servicio de Farmacología Clínica
Paseo de la Castellana, 261 CP 28046 Madrid
Teléfono +34912071466 /Fax +34912071466

Comite Cientifico

Servicio de Farmacología Clínica
Dr. Antonio J. Carcas
Dra. Irene García García
Dra. Amelia Rodríguez Mariblanca
Dra. Lucía Martínez de Soto
Dra. María José Rosales
Servicio de Cuidados Intensivos Pediátricos
Dr. Pedro de la Oliva Senovilla
Dr. Miguel Rodríguez Rubio
Servicio de Medicina Interna. Enfermedades Infecciosas
Dr. José Ramón Arribas
Dr. Juan González

Centros participantes

Centro	Investigador Principal
**Hospital Universitario La Paz**	Juan González
**Hospital Ramón y Cajal**	Begoña Monge
**Hospital Infanta Sofía**	Pilar Rúiz
**Hospital 12 de Octubre**	Antonio Lalueza
**Hospital Clínico San Carlos**	Vicente Estrada
**Hospital Central de la defensa Gómez Ulla**	Javier Membrillo
**Hospital de La Princesa**	Jesus Sanz
**Hospital Infanta Leonor**	Guillermo Cuevas

Podrán adherirse más hospitales nacionales, previa solicitud de autorizacion al CEIm y AEMPs.

Identificación del monitor.

Personal de la Plataforma Española de Ensayos Clínicos (SCReN)

Comité de Ética evaluador

El estudio será evaluado por el Comité de Ética de la Investigación con medicamentos (CEIm) del Hospital Universitario La Paz acreditado por el Ministerio de Sanidad y por las autoridades sanitarias de la Comunidad Autónoma de Madrid.

Duración prevista del ensayo

La duración de estudio será la de la pandemia por COVID19 en las áreas geográficas de los centros participantes.

ANTECEDENTES Y JUSTIFICACIÓN

INTRODUCCIÓN

El 31 de diciembre de 2019, la Comisión Municipal de Salud y Sanidad de Wuhan (provincia de Hubei, China) informó a la Organización Mundial de la Salud sobre un grupo de 27 casos de neumonía de etiología desconocida, con una exposición común a un mercado mayorista de marisco, pescado y animales vivos en la ciudad de Wuhan, incluyendo siete casos graves. El agente causante de esta neumonía fue identificado como un nuevo virus de la familia Coronaviridae que posteriormente se ha denominado SARS-CoV-2.[1] El cuadro clínico asociado a este virus se ha denominado COVID-19.

El primer caso en España fue reportado el día 31 de Enero de 2020 y el primer fallecido a causa de COVID-19, se reportó el 13 de febrero de 2020, cuando el total de casos en España era aún de 3 casos confirmados. Ante el crecimiento exponencial de casos confirmados, el 14 de Marzo de 2020 fue declarado el estado de Alarma, con una cifra que ascendía a 6.332 casos de los cuales 517 se habían recuperado, pero 193 habían fallecido. A fecha 25 de marzo de 2020 se han detectado 375.498 casos a nivel global y el número de fallecidos asciende a 17.877. En España, en esta misma fecha, el número de casos confirmados asciende a 47.610 con un total de 3.434 fallecidos. [2]

El número de nuevos casos continúa actualmente en aumento. COVID-19 es una emergencia de salud pública internacional. La emergencia de un nuevo virus, hasta el momento desconocido obliga a tomar medidas teniendo en cuenta el conocimiento científico existente con virus y situaciones similares previas. La comunidad científica mundial ha generado gran cantidad de información que se irá modificando a medida que se obtengan nuevas evidencias.

Fuente de infección y transmisión

Los coronavirus son una familia de virus que causan infección en los seres humanos y ciertos animales, incluyendo aves y mamíferos como camellos, gatos y murciélagos. Al tratarse de una enfermedad zoonótica, pueden transmitirse de los animales a los humanos. [1]

Entre humanos, la vía de transmisión se produce a través de las secreciones de personas infectadas, principalmente por contacto directo con gotas respiratorias, que pueden transmitirse a distancias de hasta 2 metros, y las manos o los fómites contaminados con estas secreciones seguido del contacto con la mucosa de la boca, nariz u ojos. La transmisión aérea o por aerosoles (capaz de transmitirse a una distancia de más de 2 metros) no ha podido ser demostrada en el brote de SARS-CoV-2 en China, sin embargo, se cree que esta podría ocurrir durante la realización de procedimientos médicos invasivos del tracto respiratorio.

El periodo de incubación medio es de 5-6 días, con un rango de 0 a 24 días. El tiempo medio desde el inicio de los síntomas hasta la recuperación es de 2 semanas cuando la enfermedad ha sido leve y 3-6 semanas cuando ha sido grave o crítica. [1] La progresión desde los primeros síntomas leves de la enfermedad a cuadros mas graves con afectación respiratoria y síndrome de distrés respiratorio se debe probablemente a la liberación incontrolada de citokinas. [3] El tiempo medio desde que aparecen los primeros síntomas hasta el desarrollo de síndrome de distrés respiratorio es de 8 días (rango intercuartílico 6-12 días) y de 2-8 semanas hasta que se produce el fallecimiento. [1,4] El promedio de casos secundarios producidos a partir de un caso se ha estimado entre 2-3[1]

El genoma del virus se detecta, por lo general, desde el inicio de los síntomas, alcanza su pico máximo entre los días 5 y 6, y baja significativamente o desaparece sobre el día 10. No obstante, en algunos casos se han detectado cantidades pequeñas de genoma alta carga viral en las muestras nasofaríngeas o de saliva, se ha concluido que este es uno de los factores que probablemente influye en la alta transmisibilidad de este virus. [1]

Uno de los colectivos más afectados por la pandemia es el personal sanitario. La afectación del personal sanitario por SARS-CoV-2 es especialmente relevante por varios motivos: (1) donde la incidencia de COVID-19 es elevada, se requiere la participación de personal sanitario para poder atender las necesidades asistenciales que se presentan; (2) la baja laboral y la necesidad de aislamiento durante 14 días merma los ya escasos recursos humanos disponibles para atender la epidemia; (3) además, el personal sanitario que desarrolla enfermedad asintomática puede transmitir de modo inadvertido la enfermedad a los pacientes que atiende, contribuyendo de este modo a mantener la epidemia en población más vulnerable.

Existe una importante falta de conocimiento respecto a potenciales intervenciones que puedan prevenir el desarrollo de COVID-19. La identificación de estas intervenciones de manera rápida podría prevenir el desarrollo de la enfermedad en personas expuestas al virus cambiando la evolución de la pandemia y evitando el consumo de recursos personales, logísticos y farmacológicos valiosos en época de alta sobrecarga del sistema sanitario.

El proyecto propuesto parte de las intensas actividades asistenciales de los hospitales participantes y tiene por objetivo identificar con rapidez estrategias preventivas eficaces para la infección por SARS CoV 2.

La baja tasa de infección grave en niños y adultos jóvenes parece indicar que condiciones propias de estas edades sean factores protectores frente a COVID-19, es por ello que este estudio busca evaluar la eficacia y seguridad de la melatonina como prevención frente a la enfermedad.

JUSTIFICACIÓN DEL USO DE MELATONINA EN PROFILAXIS

La infección por SARS-CoV2 **respeta de manera llamativa a los pacientes de edad pediátrica,** ocurrió algo similar en la infección por SARS-CoV1 **[5]**.

Según el CDC-China de 44.762 casos, 1% eran < 10 años y 1% entre 10 y 19 años y no hubo ningún fallecimiento en <10 años vs. 8% en 70-79 años y 14,8% > 80 años[6]. En Wuhan de una cohorte de 171 casos confirmados en niños únicamente ingresaron 3 pacientes pediátricos (1,7%) en la UCIP[7]. En España e Italia la distribución está siendo similar. En Madrid solo hemos registrado 3 pacientes pediátricos con Covid19 (fallo respiratorio agudo) que hayan requerido ingreso en la UCIP.

Por otro lado, el COVID19 es más frecuente y grave en varones[8] y en pacientes con comorbilidades, enfermedad cardiovascular, hipertensión arterial o diabetes[9].

Aunque la razón de estas diferencias no se conoce, es relevante reseñar que la infección por SARS-CoV-2 tiene predilección por los grupos de pacientes que exhiben como factor común la **menor producción de melatonina**: edad mayor a 50 años y en especial mayores 60 años,[10,11] varones,[12] y pacientes en los que la **melatonina** está de alguna manera involucrada en la enfermedad como son las enfermedades endocrino-metabólicas[13] y cardiovasculares.[14, 15]

Adicionalmente, la patogénesis de COVID19 se caracteriza por: inflamación excesiva (aumento de Proteína C reactiva, IL6 mioglobina, troponina, dinero-D), **oxidación y respuesta inmune exagerada que conduce a una tormenta de citoquinas y a fallo multiorgánico, principalmente Síndrome de Distress Respiratorio agudo** (SDRA)[16] pero también fallo cardio-vascular, renal y cerebral[17]

En sangre de pacientes con COVID-19 se ha observado un marcado incremento de interleukina1β (IL-1β), interferon γ (IFN-γ), interferon protein inducible 10 (IP-10), y proteína quimioatrayente de monocitos 1 (MCP-1), así como IL-4 e IL-10. Como ya hemos dicho, se ha visto también una potencial respuesta inmune deprimida en los pacientes con COVID-19, que presentan hipoalbuminemia, linfopenia, neutropenia y un porcentaje de linfocitos T CD8+ disminuido. En este sentido, la melatonina, como potente antioxidante, antiinflamatorio e inmunomodulador, podría prevenir el desarrollo de COVID19 grave. [16]

Shiu y cols[18] fueron los primeros en recomendar la **melatonina** frente a la infección por coronavirus. En ese momento el SARS-Cov1 había infectado a 5.050 personas con 316 fallecidos en 26 paises. Argumentaban que, debido a sus propiedades como **potente antioxidante[19-22]** con **actividad antiinflamatoria[23]**, sus efectos protectores frente **a infecciones virales** demostradas en animales[24-26] y los efectos positivos de la **melatonina** frente a **condiciones clínicas con fisiopatología similar al SARS**[27], merecía

la pena usar la **melatonina** en conjunción con otras terapias para tratar el SARS con la intención de incrementar la eficiencia de los fármacos convencionales y **reducir la tasa de mortalidad**[28]. Defendían que La melatonina es barata y ha **exhibe un muy alto margen de seguridad** y podría ofrecer un relevante beneficio para mejorar el estado clínico y reducir la muerte en pacientes con SARS.

Una década más tarde, con la llegada de la epidemia de Ébola, tres grupos de investigación diferentes recomendaron el uso de la **melatonina** para tratar el Ébola utilizando argumentos similares a los esgrimidos por Shiu.[29-31]En ese momento se había mostrado que en humanos la **melatonina** reducía el **estado protrombótico**[32] y que junto al SB-73 era **superior al aciclovir** frente al herpes-1[33].

Se han detectado altos niveles de melatonina en la ***mitocondria*** que es el lugar donde se producen los **radicales libres** y donde se impone el **estrés oxidativo**[37]. La **melatonina neutraliza los ROS/RNS** vía acciones independientes de receptor y por tanto reducen el **daño *mitocondrial*** y la **cascada apoptótica celular**[38]. En este sentido, se ha encontrado que la **melatonina** podría **proteger las *mitocondrias* pulmonares** de los efectos del envejecimiento y se ha propuesto como un tratamiento excelente para combatir el estado hiperoxidativo propio de los pulmones envejecidos y para **prevenir las complicaciones respiratorias** en el anciano.[39]

Por otro lado, el inflamosoma NLRP3 es un componente intracelular del sistema de respuesta inmune innato que conduce a la secreción de citoquinas proinflamatorias IL-1 β /IL-18 y a apoptosis y que se activa en respuesta a la infección bacteriana, fúngica o viral o a daño celular.[40] Sin embargo, la activación aberrante o disregulada del inflamosoma conduce a un estado de ***inflamación patológica*** con el consiguiente daño tisular y **disfunción/fallo de órganos vitales**. Es relevante que la **melatonina** tiene **efectos protectores frente a los procesos inflamatorios** modulando la activación del NF-κB (inductor de interleucinas como la IL-6) y del NLRP3 inflamosoma[41]. La actividad antiinflamatoria de la **melatonina** ha sido demostrada en humanos.[42, 43]

En resumen, la **melatonina** tiene una plétora de acciones que la hacen extraordinariamente eficaz en **reducir la agitación subcelular inducida por la destrucción oxidativa de elementos intracelulares clave**, los cuales, cuando dañados, comprometen las funciones de las células resultando en su desintegración vía apoptosis o necrosis conduciendo a un **estado inflamatorio y fallo multiorgánico.**[38]

La **melatonina** parece apuntar directamente a los **eventos inmuno-inflamatorios** asociados con la infección grave por SARS CoV-2 que afectan de forma selectiva a grupos afectados por el proceso de **envejecimiento**, **con reducida producción de melatonina** y/o **elevado estrés oxidativo y disfunción mitocondrial y ha mostrado ser un tratamiento eficaz en las infecciones virales**[44] **y en la sepsis**[27, 45] .

Además, recientemente se ha identificado que la **melatonina** regula indirectamente la expresión del *ACE2*, *un receptor clave involucrado en la infección por coronavirus*, entre ellos 2019-nCoV/SARS-CoV-2. De manera mas concreta se ha descrito que la melatonina inhibe la calmodulina, la calmodulina interactúa con ACE2 en un proceso clave para la infección por SARS-COV, de este modo la **melatonina podría inhibir indirectamente la infección del SARS-CoV2**[46, 47].

En resumen, la **melatonina** inhibiría indirectamente el proceso clave de la infección del coronavirus y además modularía y neutralizaría los efectos celulares negativos desencadenados por la infección del SARS-CoV19 evitando o minimizando la gravedad propia del Covid19 en los adultos.

Lo que se pretende con el uso preventivo de la **melatonina** es transformar la enfermedad Covid19 grave propia de los adultos en infecciones asintomáticas o leves propias del Covid19 en los niños.

El objetivo de nuestro estudio es llevar el nivel del pico nocturno de melatonina al rango de los niños menores de 10 años (> 100 pg/mL). [11,48]Las dosis de melatonina utilizada en humanos oscilan entre 0,1 y 50 mg/Kg. [49] Dosis <0,5 mg no garantiza que todos los individuos alcancen el nivel. [50] Dosis superiores a 2 mg generan picos suprafisiológicos que pueden persistir durante el periodo diurno y generar somnolencia.[51] Dosis entre 0,5 y 2 mg garantizarían que todos los individuos alcancen el nivel de melatonina nocturno >100 pg/mL, independientemente de sus niveles basales.

Existe la necesidad urgente de evaluar mediante ensayos clínicos las potenciales intervenciones que puedan prevenir la infección por SARS-CoV-2 en personal sanitario con alto riesgo de contagio. La melatonina se postula como un potencial candidato por su actividad antiinflamatoria, antioxidante y protectora, en infecciones bacterianas y virales,12 además, inhibe indirectamente un proceso clave de la infección por coronavirus. De este modo se postula que podría llegar a evitar la infección además de que tener potencial para modular y neutralizar los efectos celulares negativos desencadenados por la infección por SARS-CoV19 evitando o minimizando la gravedad propia del COVID19 en los adultos.

De manera adicional a todo lo anteriormente expuesto, melatonina es un producto barato y accesible con muy escasos efectos adversos.

El diseño del estudio es un ensayo clínico multicéntrico abierto, aleatorizado y controlado con placebo que tiene por objetivo principal evaluar la eficacia de melatonina en la prevención de la infección por SARS-CoV-2 en personal hospitalario con contactos de alto riesgo.

PLANTEAMIENTO DEL ESTUDIO

Diseño de ensayo.

El diseño del estudio es un ensayo clínico multicéntrico, aleatorizado doble ciego y controlado con placebo que tiene por objetivo principal evaluar la eficacia de melatonina en la prevención de la infección por SARS-CoV-2 en personal hospitalario con contactos de alto riesgo.

Hipótesis

La administración de melatonina previene el desarrollo de infección por SARS-CoV-2 en contactos de alto riesgo (personal hospitalario).

Objetivos.

Objetivo principal:

- Evaluar la eficacia de melatonina en la prevención de la infección sintomática por SARS-CoV-2 en personal hospitalario con contactos de alto riesgo.

Objetivos secundarios:

- Evaluar la eficacia de melatonina en la prevención de infecciones asintomáticas por SARS-CoV-2 (COVID-19).
- Evaluar la eficacia de melatonina en la prevención del desarrollo de COVID-19 grave en participantes que adquieran la infección durante el desarrollo del estudio.
- Evaluar la duración de los síntomas de la infección por COVID-19 en pacientes que recibieron melatonina.
- Evaluar la seroconversión de IgM/IgG desde la detección de síntomas

Objetivos exploratorios:

- Evaluar las diferencias en la gravedad de COVID-19 entre hombres y mujeres.
- Evaluar la influencia del sueño y la alimentación en la prevención del desarrollo de COVID-19.
- Evaluar el efecto de la melatonina en la incidencia y características de la linfopenia y la elevación de citoquinas inflamatorias en individuos infectados.

SELECCIÓN DE PARTICIPANTES

Se seleccionarán hombre y mujeres adultos que formen parte del personal hospitalario que directa o indirectamente estén expuestos a la infección por coronavirus en el ejercicio profesional.

Para que los resultados sean comparables a los resultados del Ensayo clínico para prevención de la Infección por coronavirus en sanitarios que está poniendo en marcha el Ministerio de Sanidad, (Ensayo clínico EPICOS) asumimos sus mismos criterios de inclusión y exclusión.

Los participantes en el estudio deben cumplir todos los criterios de inclusión señalados a continuación y ninguno de los criterios de exclusión.

Criterios de inclusión:

- Hombre y mujeres con edad entre 18 y 70 años.
- Forman parte del personal hospitalario de hospitales públicos o privados de la red hospitalaria española en una zona con riesgo de transmisión de SARS-CoV-2.
- No haber sido diagnosticado previamente de SARS-CoV-2 (COVID-19).
- Entender el propósito del estudio y NO haber tomado ninguna medicación como PrEP frente a SARS-CoV-2 desde el 1 de marzo de 2020 hasta la entrada en el ensayo (también incluye PrEP para el VIH).
- Tener una PCR o test de diagnóstico rápido IgM/IgG para SARS-CoV-2 negativo a la entrada.
- Prueba de embarazo en orina negativa realizada en los 7 días anteriores al comienzo del tratamiento en estudio en mujeres pre-menopáusicas o < 2 años después de la menopausia.
- Las mujeres en edad fértil y los varones con pareja en edad fértil deben comprometerse a utilizar un método anticonceptivo de gran eficacia (como esterilización quirúrgica, método de doble barrera, anticonceptivos orales o implantes hormonales contraceptivos) y a continuar utilizándolos hasta el díade la última dosis de tratamiento.

Criterios de exclusión:

- Infección por el VIH.
- Infección activa por virus de la hepatitis B.
- Insuficiencia renal (aclaramiento de creatinina < 60 ml/min) y pacientes en hemodiálisis.
- Osteoporosis.
- Miastenia gravis.
- Maculopatía preexistente del ojo.

- Retinitis pigmentosa.
- Bradicardia < 50bpm.
- Peso < 40kg.
- Participantes con enfermedad inmunosupresora o hematológica.
- Tratamiento en el último mes antes de la aleatorización y durante más de 7 días, con fármacos que pueden prolongar el Q, T incluidos: azitromicina, clorpromazina, cisaprida, claritromicina, domperidona, droperidol, eritromicina, halofantrina, haloperidol, lumefantrina, mefloquina, metadona, pentamidina, procaina quinidina, quinina, sotalol, esparfloxacina, tioridazina, amiodarona.
- Los participantes que presenten intolerancia hereditaria a la galactosa, de insuficiencia de lactasa de Lapp o malabsorción de glucosa o galactosa.
- Tratamiento con fluvoxamina.
- Tratamiento con benzodiacepinas o hipnóticos no benzodiacepínicos, tales como el zaleplón, el zolpidem y la zopiclona.
- Embarazo o planificación de quedarse embarazada durante el transcurso del estudio. Lactancia
- Pacientes con historia de enfermedades potencialmente mediadas por el sistema inmune o enfermedades inflamatorias: lupus eritematoso sistémico, Crohn, colitis ulcerosa, vasculitis, artritis reumatoide.
- Pacientes con diabetes mellitus insulinodependientes.
- Hipersensibilidad al principio activo o a alguno de los excipientes.
- Participantes que por cualquier motivo no deberían ser incluidos en el estudio según evaluación del equipo investigador.

Subestudio InmunoCOVID

Anidado a este ensayo clínico se realizará el ensayo clínico InmunoCOVID.

Los primeros 50 participantes incluidos en el Hospital Universitario La Paz podrán participar en un subestudio InmunoCOVID cuyo Investigador Principal es Eduardo López Granados.

Tamaño de la muestra.

En un estudio realizado en Wuhan[52] se observó que en una cohorte de sujetos con exposición de alto riesgo el 54% desarrollaba una infección por SARS-CoV-2. Calculamos que con esta intervención se conseguirá reducir un 20% el número de contagios. Para un poder estadístico de al menos el 90% y un error alfa del 5%, se necesitan aproximadamente 450 sujetos para demostrar nuestra hipótesis.

TRATAMIENTOS EN EVALUACIÓN.

El producto en investigación es Circadin® 2 mg comprimidos de liberación prolongada.

Grupos de tratamiento.

En los participantes que cumplan los criterios de selección se hará una aleatorización estratificada por sexo, edad (<50 y > o =50 años) y centro a través de la aplicación RedCAP, a uno de los dos siguientes grupos de tratamiento (1:1):

- **Grupo 1:** Melatonina 2 mg diarios, 1 o 2 horas antes de acostarse, y después de haber ingerido algún alimento durante 12 semanas.
- **Grupo 2:** Placebo de aspecto idéntico 2 mg diarios, 1 o 2 horas antes de acostarse, y después de haber ingerido algún alimento durante 12 semanas.

La hora de administración podrá adaptase en función del turno de trabajo del participante.

Los participantes con positivización de la PCR y/o serología y/o test de diagnóstico rápido para SARS-CoV-2 a lo largo del estudio pasarán a manejo según práctica clínica habitual, pero se realizará seguimiento de su evolución hasta resolución de la enfermedad o fallecimiento.

En aquellos participantes que no presenten sintomatología compatible con COVID-19 se realizará seguimiento hasta el día 30.

Tratamientos permitidos y prohibidos.

En el momento de iniciar el estudio, los participantes continuarán recibiendo todas las medicaciones concomitantes que les hayan prescrito sus médicos siempre que se considere adecuado a criterio médico. El tratamiento concomitante debe recogerse en el Cuaderno de Recogida de Datos.

Además, se deberán registrar todos los procedimientos diagnósticos, terapéuticos o quirúrgicos realizados durante el periodo del estudio, incluyendo la fecha, indicación, descripción del procedimiento y todos los hallazgos clínicos.

El equipo médico del paciente sigue siendo en todo momento responsable de las decisiones sobre la atención y seguridad de cada paciente. Por lo tanto, la administración de fármacos de estudio debe detenerse si el equipo sospecha cualquier reacción grave inesperada relacionada con los tratamientos en estudio.

Criterios de retirada y abandono.

De acuerdo con la revisión actual de la Declaración de Helsinki (Fortaleza, Brasil octubre 2013) (Anexo 2), y con la normativa aplicable, un paciente tiene derecho a retirarse del estudio en cualquier momento y por cualquier razón, sin que ello le suponga perjuicio en la atención médica por parte de su médico y/o centro de referencia en el futuro.

Los participantes son libres de retirarse del tratamiento del estudio en cualquier momento, pero todavía podrían permanecer en el estudio, y el hospital reportar el resultado final al estudio al morir o dar de alta al paciente.

Los participantes también son libres de retirarse de todo el estudio en cualquier momento sin ninguna consecuencia, y se le seguiría ofreciendo el tratamiento convencional que se le viene dando a todos los pacientes con COVID que acuden a este hospital.

Un paciente podrá ser retirado del estudio en los siguientes casos:

- El paciente retira su consentimiento.
- Por razones de seguridad: si aparecen acontecimientos adversos que por su tipo o gravedad hagan que el paciente no deba permanecer en el estudio.

En cualquier caso, las causas que hayan motivado el abandono o retirada del paciente del estudio serán detalladamente registradas en el CRD. Tanto si el paciente abandona el estudio como si se le retira a criterio del investigador, su evolución se recogerá en el CRD, así como la fecha de suspensión y la nueva terapia pautada.

Por lo demás, una vez que el paciente no esté dentro del estudio, será atendido por el médico correspondiente según la práctica clínica habitual.

DESARROLLO DEL ESTUDIO Y PROCEDIMIENTOS.

El desarrollo y evaluaciones del ensayo se pretende que se realicen de manera pragmática, ajustándose lo más posible a la práctica clínica en una situación de emergencia como la actual.

Se realizarán 4 visitas presenciales Basal (D1), Semana 4 (D30), Semana 8 y Semana 12.

Se realizará una llamada telefónica de seguimiento y fin de estudio 4 semanas tras la última toma del medicamento en estudio para preguntar por evolución, sintomatología, diagnóstico de COVID19 y acontecimientos adversos.

Evaluaciones y procedimientos del estudio.

Tabla 1: Evaluaciones y procedimientos del estudio

Calendario de evaluaciones	Visita de Screening<br>Día 1	Visita presencial S4<br>D30 (+/- 1días)	Visita presencial S8<br>S8 (+/- 5días)	Visita presencial S12<br>S12 (+/- 5días)	Llamada telefónica S16<br>S16 (+/- 15días)
Criterios de selección	x				
Consentimiento informado	x				
Historia Clínica	x	x	x	x	x
Exploración física	x	X[1]	X[1]	X[1]	
Signos vitales	x	x	x	x	
Talla y peso	x		x		
Analítica sanguínea	x	x	x	x	
Aleatorización	x				
Inicio de Medicación de estudio	x				
Administración medicación de estudio	x	x	x	x	
Registro de acontecimientos adversos	x	x	x	x	x
Registro de la medicación concomitante	x	x	x	x	x
Recordar la importancia de cumplimentar los datos en el formulario MeCOVID	x	x	x		
PCR o Test de diagnóstico rápido para SARS-CoV-2.	x				
PCR y/o serología para SARS-CoV-2 y/o test de diagnóstico rápido para SARS-CoV-2		x	x	x	
Muestra de suero para archivo[2]	x	x	x	x	
Muestra para subestudio inmunológico[*]	x	x			

[1] A juicio del investigador.
[2] Se recogerán muestras de suero en cada visita del estudio.
[*] Solo en los participantes en el subestudio.

Visitas y procedimientos.

Las visitas y procedimientos a seguir durante el estudio se describen en la tabla 1 y se detallan a continuación.

Día 1 – Screening:

Tras informar al paciente sobre el estudio, una vez firmado el consentimiento informado, y comprobar los criterios de selección se llevarán a cabo los siguientes procedimientos:

- Historia clínica detallada con datos demográficos, características basales, alergias y antecedentes médicos y quirúrgicos.
- Exploración física incluyendo signos vitales (tensión arterial, frecuencia cardíaca, temperatura, SatO2(%) basal, peso (kg) altura (m)).
- Extracción sanguínea con hematología (hemograma, recuento diferencial y plaquetas) y bioquímica (Función renal (creatinina, urea, y electrolitos, fósforo), función hepática (AST, ALT, bilirrubina total y fraccionada, GGT, LDH, fosfatasa alcalina), D-Dímero, proteína C reactiva (PCR), colesterol, triglicéridos, troponina, albúmina,glucosa y ferritina.
- Extraccion de suero de archivo del estudio.
- PCR para COVID-19 o test de diagnóstico rápido.
- Randomización (RedCAP) y entrega de medicación.
    - Antes de entregar la medicación se debe comprobar que el paciente cumple todos los criterios de inclusión y ninguno de exclusión. Podrán transcurrir un máximo de 5 días entre la realización de los procedimientos del screening y el inicio de la medicación. Se considerará Día 1 el día en el que se inicie la toma de la medicación.
- En aquellos sujetos que participen en el estudio inmunológico, se les extraerá una muestra de sangre adicional.

Visitas presenciales S4, S8, S12:

Se realizarán los siguientes procedimientos siempre y cuando sean posibles teniendo en cuenta la carga asistencial y posibilidades del centro:

- Historia clínica.
- Exploración física (a criterio del investigador).
- Signos vitales (tensión arterial, frecuencia cardíaca, temperatura, SatO2(%) basal.
- Extracción sanguínea con hematología (hemograma, recuento diferencial y plaquetas) y bioquímica (Función renal (creatinina, urea, y electrolitos, fósforo), función hepática (AST, ALT, bilirrubina total y fraccionada, GGT, LDH, fosfatasa alcalina), D-Dímero,

proteína C reactiva (PCR), colesterol, triglicéridos, troponina, albúmina,glucosa y ferritina.

- Extraccion de suero de archivo del estudio.
- En aquellos sujetos que participen en el estudio inmunológico, se les extraerá una muestra de sangre adicional en la visita de la semana 4 (día 30).
- PCR para COVID-19, y/o serología y/o test de diagnóstico rápido para COVID-19 .
- Revisión de la medicación concomitante.
- Revisión y documentación de acontecimientos adversos.

Llamadas telefónicas de seguimiento:

Se realizará una llamadas telefónicas de seguimiento 4 semanas tras la última toma de la medicación del estudio.

Durante la llamada se preguntará al participante lo siguiente:

- Aparición de nueva sintomatología o empeoramiento si presentaban algún tipo de clínica en el momento de inclusión en el estudio.
- Se les interrogará de manera dirigida sobre la aparición de acontecimientos adversos, intensidad de los mismos, necesidad de toma de medicación e o si han recibido alguna atención médica en relación al mismo.
- Se les interrogará sobre medicación concomitante.

Diario online del sujeto.

Los participantes recibirán por correo electrónico un enlace para acceder al Formulario inicial y Formulario diario diseñado para este estudio y registrarán diariamente los datos especificados en la **Tabla 2.**

Los participantes deben acceder al enlace que recibirán por correo electrónico el día de su inclusión en el estudio.

Cumplimentaran los datos solicitados desde el D1, cuando se inicie la toma de la medicación del estudio, hasta la semana 12.

Los participantes deben contactar inmediatamente con el equipo investigador si presentan nueva sintomatología o empeoramiento de sintomatología previa, así como si se les realizan pruebas complementarias en relación a una posible infección por SARS-CoV-2.

Tabla 2: Registro diario de datos en el Formulario MECOVID

**DATOS INICIALES**
-Hospital
-Número de participante
-Rama de tratamiento
-Sexo
-Edad
-Peso (en kg)
-Talla (en metros)
**REGISTRO DE DATOS DIARIA A CUMPLIMENTAR POR LOS PARTICIPANTES**
-Fecha del registro
-Hora a la que se acuesta
-Hora a la que se despierta
-Temperatura axilar por la mañana (ºC)
-Temperatura axilar por la tarde (ºC)
-SatO basal (%)
-Número de cigarrillos
-Minutos de exposición a luz natural (tiempo al aire libre mientras es de día)
-Minutos de ejercicio vigoroso (Ejemplo: correr, bicicleta, aerobic)
-Minutos de actividad física moderada (Ejemplo: caminar a paso rápido, pasear)
-Registro de la ingesta de alimentos ricos en melatonina: Plátanos (unidades)
-Registro de la ingesta de alimentos ricos en melatonina: Nueces (puñados)
-Registro de la ingesta de alimentos ricos en melatonina: Tomates (unidades)
-Registro de la ingesta de alimentos ricos en melatonina: Arroz (puñados)
-Registro de la ingesta de alimentos ricos en melatonina: Maíz (puñados)
-Registro de la ingesta de alimentos ricos en melatonina: Avena(puñados)
-Aparición de cefalea, hora de inicio, intensidad.
-Aparición de mareo, hora de inicio, intensidad.
-Aparición de somnolencia, hora de inicio, intensidad.
-Aparición de naúseas, hora de inicio, intensidad.
-Número de horas trabajadas en tuno de noche
-Registro de otros acontecimientos adversos, hora de inicio, intensidad.
-Horas dormidas durante periodo diurno (mientras hay luz natural) Ejemplo: siesta
-Medicamentos concomitantes diferentes a tratamiento habitual señalado en la visita de screening
-Grado de exposición a infección por SARS-2-CoV 19: Clasificado como: Alto riesgo/ Bajo riesgo/ No exposición.**
- ¿Ha tomado la medicación? Hora a la que ha tomado la medicación

** Se medirá el grado de exposición a infección por SARS-2-CoV mediante el cumplimiento en el Formulario MeCOVID de los datos de exposición, que se clasificará en alto o bajo riesgo, según las definiciones establecidas por el Ministerio de Sanidad[53]:

1. Se considera una exposición laboral de **alto riesgo** a aquellas situaciones laborales en las que:
    a. se han realizado procedimientos que generen aerosoles como aspiración del tracto respiratorio, intubación o broncoscopia o maniobras de reanimación **sin el equipo de protección adecuado.**
    b. **contacto estrecho** con caso posible, probable o confirmado de COVID-19 **sin EPI.**
    c. **contacto casual con** caso posible, probable o confirmado de COVID-19 en unidades con pacientes especialmente vulnerables como por ejemplo hematología, UCI, oncología o unidades de quemados.
2. Se considera exposición laboral **de bajo riesgo** aquellas situaciones laborales en las que:
    a. la relación que se pueda tener **con un caso probable o confirmado no incluye contacto estrecho**.
    b. **contacto estrecho** con caso posible, probable o confirmado de COVID-19 **con uso adecuado del EPI.**

Aquellos participantes con ≥3 días con registro de alto riesgo serán categorizados a efectos de análisis como ALTO RIESGO.

Procedimientos adicionales: Individuos que resulten infectados por SARS-Cov-2

Los individuos que resulten infectados por SARS-Cov-2SARS dejarán de tomar la medicación del estudio en el momento en el que se confirme el diagnóstico y pasarán a manejo según práctica clínica habitual. Se realizará seguimiento de su evolución hasta resolución de la enfermedad o fallecimiento y se recogerán los siguientes datos de la Historia Clínica:

- Registro de PCR y/o serología que confirma infección por SARS-Cov-2.
- Ingreso hospitalario.
- Duración en días de la estancia hospitalaria.
- Escala NEWS (Anexo 1).
- Escala de los 7 puntos (Anexo 2).
- Soporte ventilatorio: Gafas nasales; Dispositivos de oxígeno de alto flujo; Marcarilla con reservorio; Ventilación no invasiva; Ventilación mecánica invasiva ECMO; Ventilación mecánica no invasiva
- Ingreso en UCI.
- Mortalidad por cualquier causa.
- Datos relacionados con la gravedad y evolución de COVID-19.

- Fenómenos tromboticos.
- Anticoagulación.

Para evitar la inclusión de personas no susceptibles, se realizarán test rápidos de SARS-2-CoV tipo IgM/IgG, que actualmente hay disponibles, a las personas que no refieran tener un diagnóstico confirmado previo. Se tendrá en cuenta la sensibilidad y especificidad de los mismos. Los casos positivos serán excluidos del ensayo. Si bien la aleatorización garantizaría una distribución similar en los 4 grupos, se realizarán análisis de análisis de respuesta serológica en las muestras de archivo en una fase posterior..

Seguimiento.

Los participantes con positivización de la PCR y/o serología y/o del test de diagnóstico rápido para SARS-CoV-2 a lo largo del estudio pasarán a manejo según práctica clínica habitual, pero se realizará seguimiento de su evolución hasta resolución de la enfermedad o fallecimiento. En aquellos participantes que no presenten sintomatología compatible con COVID-19 se realizará seguimiento hasta 4 semanas después de la última toma de melatonina.

CRITERIOS DE EVALUACIÓN: VARIABLES EN EL ESTUDIO

Variable Principal.

- Número de infecciones sintomáticas confirmadas por SARS-CoV-2 (COVID-19).

Variable secundaria:

- Número de infecciones sintomáticas confirmadas por SARS-CoV-2 (COVID-19) estratificado por contacto de alto o bajo riesgo.
- Número de infecciones asintomáticas confirmadas por SARS-CoV-2 (COVID-19) medidas por serología.
- Número de infecciones asintomáticas confirmadas por SARS-CoV-2 (COVID-19) medidas por serología estratificado por contacto de alto o bajo riesgo.
- Gravedad de la infección por SARS-CoV-2 (COVID-19) medida en relación con lo siguiente:
  - Asintomático
  - Síntomas leves definidos como malestar general, fiebre, tos, artromialgias
  - Síntomas moderados. Los anteriores más dificultad respiratoria moderada que requiera ingreso hospitalario.
  - Síntomas graves. Los anteriores más dificultad respiratoria franca que precise medidas de UCI
- Duración de los síntomas de la infección por coronavirus COVID-19 medida en días.
  - Fiebre, mialgia, astenia, dificultad respiratoria
- Relación entre la duración en días del tratamiento y la aparición de síntomas.
- Relación entre la duración en días del tratamiento y la duración de síntomas.
- Evaluar la seroconversión de IgM/IgG desde la detección de síntomas.
- Tiempo hasta PCR y/o serología positiva para SARS-CoV-2
- Tiempo (días) hasta aparición de sintomatología compatible con COVID-19.

En aquellos participantes que a lo largo del estudio presentan PCR y/o serología y/o test de diagnóstico rápido positivo para SARS-CoV:

- Necesidad de ingreso hospitalario
- Duración en días de la estancia hospitalaria.
- Tiempo (días) hasta alta hospitalaria o hasta obtención de una puntuación en la escala NEWS (Anexo 1) ≤2 mantenido durante 24 horas, lo que ocurra antes.
- Puntuacion en la escala de 7 puntos a lo largo de la evolucion del paciente.
- Diferencia en la puntuación en la escala NEWS entre el día 1 y los días 3, 5, 8, 11, 15 y 29 de ingreso.
- Porcentaje de pacientes que requieren Oxígenoterapia.

- Porcentaje de pacientes que requieren ventilación mecánica no invasiva
- Porcentaje de pacientes que requieren ventilación mecánica invasiva.
- Porcentaje de pacientes que requieren cuidados intensivos.
- Estancia media en UCI (días)
- Mortalidad por cualquier causa.
- Progresión a COVID grave (alguna de las siguientes): 1) Progresión radiológica O 2) Necesidades de soporte ventilatorio 3) Síndorme de distrés respiratorio agudo. 4) Ingreso en UVI, o 5) Fallecimiento por COVID-19.
- Porcentaje de pacientes que sufren fenómenos tromboticos.
- Porcentaje de pacientes que reciben anticoagulación.

Otras variables de interés:

- Porcentaje de hombres y mujeres con positivización de la PCR y/o serología para SARS-CoV-2 a lo largo del estudio.
- Porcentaje de hombres y mujeres con desarrollo de COVID-19 grave.

Variables exploratorias, que se recogerán mediante un diario electrónico (APP en el móvil) que ha sido diseñado para este estudio.

Estas variables incluyen factores relacionados con los niveles fisiológicos de melatonina. Se recogen en la **Tabla 2.**

Variables inmunológicas.

Se recogerán en los primeros **50 participantes incluidos en el Hospital Universitario La Paz, para realización del subestudio.**

Incluirá: -Estudio de IL-6, -Estudio de subpoblaciones linfocitarias mediante cartometría de flujo mutiparamétrica: Th (CD4+), Tc (CD8+) incluyendo subpoblaciones näive, memoria central, efectoras y memoria efectoras

SEGURIDAD DEL ESTUDIO

Información de seguridad de deferencia

En este estudio la información de seguridad de referencia es la ficha técnica de Circadin 2 mg comprimidos de liberación prolongada.
Todos los acontecimientos adversos que ocurran, incluyendo los no graves y no relacionados con la medicación, serán resumidos en el informe final del ensayo clínico. Una vez finalizado el estudio, una copia de este informe será enviado al **sistema de farmacovigilancia de Exeltis** (Farmacovigilancia_spain@exeltis.com).

Definiciones

ACONTECIMIENTO ADVERSO (AA))

Es cualquier suceso médico adverso que pueda presentar un paciente o sujeto de investigación clínica al que se ha administrado un producto farmacéutico y que no tiene necesariamente que tener una relación causal con dicho tratamiento.

Se registrará como acontecimiento adverso:

- Empeoramiento significativo o inesperado de la condición a tratar durante el estudio.
- Exacerbación de enfermedades crónicas, intermitentes o episódicas pre-existentes, incluyendo un aumento en la frecuencia y/o la severidad de las mismas.
- Una nueva condición detectada o diagnosticada después de la administración del producto en investigación, incluso si pudiera haber estado presente antes de iniciar el estudio.
- Signos clínicos, síntomas o secuelas sospechosas.
- Signos clínicos, síntomas o secuelas de una sospecha de sobredosis del producto medicinal en investigación o de la medicación concomitantes (la sobredosis en sí misma no debe reportarse como un AA/AAG).
- Fallo obvio del efecto biológico o farmacológico esperado.
- Cualquier reacción médica antes, durante o después del tratamiento resultado de algunos de los procedimientos especificados en el protocolo como por ejemplo derivadas de la toma de muestras.

No se registrará como acontecimiento adverso:

- Procedimientos médicos o quirúrgicos debidos al curso de la enfermedad permitidos en el ensayo clínico.
- Situaciones para evitar una experiencia médica no deseada como por ejemplo la admisión en el hospital por razones sociales o de conveniencia.

- Signos clínicos, síntomas o secuelas propios de la enfermedad de estudio o la progresión esperada de la misma.

Acontecimiento Adverso Grave (AAG)

Un acontecimiento (experiencia) adverso grave es aquel suceso médico etiquetado como tal que, independientemente de la dosis:

- Produce la muerte.
- Pone en peligro la vida.
- Precisa de ingreso hospitalario o lo prolonga.
- Produce una discapacidad/incapacidad persistente o importante, o resulta en una anomalía o defecto congénito.
- Sucesos no incluidos en los párrafos anteriores pero que puedan poner en peligro al paciente o requerir intervención para prevenir cualquiera de los resultados anteriormente mencionados. Ejemplos de tales acontecimientos son el tratamiento intensivo en un servicio de urgencias o en domicilio por un broncoespasmo alérgico; las discrasias sanguíneas o convulsiones que no den lugar a ingreso hospitalario, o el desarrollo de dependencia o de abuso de fármacos.

Reacción Adversa (RA)

Una RA es toda reacción nociva y no intencionada a un medicamento en investigación, independientemente de la dosis administrada.

Se registrará como Reacción adversa grave (RAG): Cualquier reacción adversa que ocasione la muerte, pueda poner en peligro la vida, exija la hospitalización del paciente o la prolongación de la hospitalización ya existente, ocasione una discapacidad o invalidez significativa o persistente o constituya una anomalía congénita o defecto de nacimiento. A efectos de su notificación, se tratarán también como graves aquellas sospechas de reacción adversa que se consideren importantes desde el punto de vista médico, aunque no cumplan los criterios anteriores, como las que ponen en riesgo al paciente o requieren una intervención para prevenir alguno de los desenlaces anteriores. Así mismo, a efectos de su notificación, se tratarán como graves todas las sospechas de transmisión de un agente infeccioso a través de un medicamento.

Se registrará como Reacción Adversa Grave Inesperada (RAGI): Cualquier reacción adversa grave cuya naturaleza, gravedad o consecuencias no sean coherentes con la información disponible sobre el producto.

Evaluación de la causalidad.

La **evaluación de causalidad** de cada acontecimiento adverso con la medicación del ensayo lo realizará los investigadores clínicos del estudio de acuerdo a las siguientes definiciones:

- **AA relacionado**: La relación temporal del AA con la medicación en estudio indica una relación causal posible y no puede ser explicado por factores tales como el estado clínico del paciente o intervenciones terapéuticas.
- **AA no relacionado**: La relación temporal del AA con la medicación del estudio indica una relación causal improbable, o bien otros factores (medicación o condiciones concomitantes), otras intervenciones terapéuticas proporcionan una explicación satisfactoria para el AA.
- **AA inesperado:** Cualquier Acontecimiento Adverso que no ha sido previamente observado ni reportado en el manual del investigador.

Evaluación de la intensidad.

La **intensidad** de los acontecimientos adversos se clasificará en grados, en una escala de tres puntos (leve, moderada, grave) y se registrará detalladamente de la manera indicada en el CRD.

- **Leve:** Se observa molestias, pero éstas no alteran la actividad diaria normal.
- **Moderada:** Molestias suficientes para reducir o afectar la actividad diaria normal.
- **Grave o severa:** Incapacidad para trabajar o desempeñar la actividad diaria normal.

Información sobre acontecimientos adversos (AAs).

El equipo investigador hará el seguimiento y recogerá sistemáticamente los posibles AAs desde la firma del consentimiento informado y hasta la visita final de seguimiento de cada paciente.Los AA que aparezcan en el período de seguimiento a largo plazo serán publicados y vinculados al ensayo clínico.

Todos las AA que ocurran durante el estudio, hasta finalizar el período de seguimiento, sean atribuidos o no a la medicación del estudio, serán evaluados por el investigador y registrados con detalle en el CRD. Se registrarán datos de descripción del AA, fecha de inicio y fin, gravedad/intensidad, evaluación de causalidad con el medicamento de investigación, la evolución, el desenlace del AA y las medidas adoptadas (tratamientos, exploraciones complementarias adicionales) incluidas las adoptadas con respecto al medicamento en investigación (ej: suspensión). Todos los AA independientemente de la relación de causalidad, serán seguidos hasta su resolución o hasta que se considere

estable. Se dejará a criterio del investigador si considera que el AA sea de suficiente gravedad como para requerir la retirada del paciente del tratamiento. Los participantes también podrán retirarse voluntariamente si perciben como AA no tolerable. Se entregará a todos los participantes una tarjeta de participación en el estudio, con un número de teléfono para contactar con los investigadores en caso de producirse algún AA.

El desenlace de los AAs, será evaluado de la siguiente forma:

- Resuelto
- Mejoría
- Sin cambios
- Empeoramiento
- Muerte

Y la actitud tomada con respecto a la medicación de estudio:

- Ninguna;
- Abandono;
- Interrupción;

Embarazos

Las mujeres en edad fértil deben utilizar un método anticonceptivo adecuado mientras dure el estudio y hastael día de la última dosis del tratamiento em estudio.. Se indicará que a las mujeres que participan en el estudio o la pareja del hombre que participa en el estudio, en caso de quedar embarazada, deben informar inmediatamente de ello al investigador. En tal caso, se suspenderá de inmediato la medicación.

Cada embarazo que ocurra deberá ser notificado al promotor o a quien asuma las tareas delegadas por el promotor en un plazo de 24 horas desde su conocimiento. El embarazo deberá seguirse para determinar el resultado, incluyendo finalización espontánea o voluntaria, detalles del nacimiento, y la presencia o ausencia de cualquier defecto como anomalía congénita o complicaciones para la madre y/o recién nacido. Los resultados del embarazo deben registrarse también para las mujeres parejas de un paciente hombre que esté participando en el estudio. Si el desenlace del embarazo cumple criterios de AAG o si el recién nacido presenta un AAG se seguirán los procedimientos descritos para la notificación de AAG.

Procedimiento para la notificación de los acontecimientos adversos graves

En caso de que se produzca un AAG, el investigador lo comunicará al promotor o a quien

asuma las tareas delegadas por el promotor en un plazo máximo de 24 horas a partir del momento en que tenga conocimiento de él.

Así mismo, el investigador cumplimentará y firmará el formulario de notificación de AAG que se enviará por fax o correo electrónico a:

Responsable de Farmacovigilancia
Dra. Irene García García
Unidad de Ensayos Clínicos
Hospital Universitario La Paz
Paseo de la Castellana, 261
28046 Madrid
irene.ucicec@gmail.com
Teléfono +34912071466 /Fax +34912071466

El promotor o quien asuma las tareas delegadas por el promotor revisará el formulario recibido y, si procede, solicitará información adicional al investigador. El investigador proporcionará información al promotor o a quien asuma las tareas delegadas por el promotor siempre que se le solicite y, en cualquier caso, cuando cambie su evaluación inicial en cuanto a gravedad o causalidad. Para comunicar la información de seguimiento se seguirá el procedimiento de notificación descrito previamente.

El promotor o quien asuma las tareas delegadas por el promotor llevará un registro detallado de todos los AAG que le sean comunicados por los investigadores.

Procedimiento para la notificación de los acontecimientos adversos graves e inesperados (RAGI)

El promotor o quien asuma las tareas delegadas por el promotor, notificara todas las sospechas de RAGI de acuerdo a la normativa vigente sobre ensayos clinicos a la Agencia Espanola de Medicamento y Productos Sanitarios (AEMPS), a los comites eticos (CEIC), a las autoridad sanitaria de la Comunidad Autonoma de Madrid, y al sistema de farmacovigilancia de Exeltis Healthcare (a través del correo-e: Farmacovigilancia_spain@exeltis.com) en un plazo máximo de quince (15) días naturales a partir del momento en el que se tenga conocimiento de las mismas. Cuando la RAGI haya ocasionado la muerte del paciente o puesto en peligro su vida, la notificación se realizara en un plazo máximo de siete (7) días naturales a partir del momento en el que se tenga conocimiento de la misma. Se complementara la información pertinente relativa a los hechos posteriores en un plazo de ocho (8) dias.

La información mínima inicial para la notificación de un acontecimiento adverso

debe incluir lo siguiente:

- Acontecimiento adverso y fecha de comienzo del mismo.
- Iniciales, sexo y edad (o fecha de nacimiento) del paciente.
- Información sobre el tratamiento recibido.
- Nombre y dirección del médico que realiza la notificación.
- Relación de causalidad con la medicación en estudio

ASPECTOS ETICOS

Normas generales y particulares para los investigadores.

Los investigadores se atendran estrictamente a lo dispuesto en este protocolo, cumplimentando totalmente las hojas del cuaderno de recogida de datos. El estudio se llevara a cabo de acuerdo a las recomendaciones para Estudios Clinicos y evaluacion de farmacos en humanos, que figuran en la ultima version de la Declaracion de Helsinki (Fortaleza, Brasil Octubre 2013, Anexo 2), revisada en las sucesivas asambleas mundiales, y la actual Legislacion Espanola en materia de Estudios Clinicos (Real Decreto 1090/2015 de 4 de diciembre y el Reglamento Europeo 536/2014 de 16 de abril, por los que se regulan los ensayos clinicos con medicamentos).

Todos los participantes serán informados antes de iniciar el estudio de los objetivos, procedimientos e incomodidades relacionadas con los mismos, así como de los riesgos del estudio según una hoja de información.

En cualquier momento, el paciente podrá revocar el consentimiento informado para el tratamiento de sus datos personales, dirigiéndose directamente al investigador.

Dispositivos de seguridad y confidencialidad.

La información difundida y obtenida por la puesta en marcha del presente estudio es considerada confidencial y deberá ser tratada en todo momento como tal.

La confidencialidad de los datos personales obtenidos estará amparada, respetando en todo momento los principios éticos básicos de la investigación con muestras biológicas, y lo establecido por la legislación aplicable, básicamente Reglamento (UE) 2016/679 del Parlamento europeo y del Consejo de 27 de abril de 2016 de Protección de Datos (RGPD), la Ley 41/2002 reguladora de la Autonomía del Paciente y de los Derechos y Obligaciones en Materia de Información y Documentación Sanitaria y la Ley 14/1986 General de Sanidad.

Seguro:

Según lo dispuesto en el RD1090/2015 del 4 de diciembre por el que se regulan los ensayos clínicos con medicamentos, este estudio se plantea como “Ensayo clínico de bajo nivel de intervención” dado que:

1. El medicamento en investigación está autorizado en España.
2. Según este protocolo de ensayo clínico: La utilización de los medicamentos se basa en datos científicos publicados sobre su eficacia y seguridad.

3. Los procedimientos complementarios de diagnóstico o seguimiento entrañan un riesgo o carga adicional para la seguridad de los sujetos que es mínimo comparado con el de la práctica clínica habitual, dado que únicamente implica la extracción de una muestra de sangre y/o toma de muestras respiratorias de exudado nasofaríngeo para realización de PCR para SARS-CoV-2. El resto de procedimientos que pueden realizarse forman parte de la práctica clínica habitual.

Justificación: Como argumento adicional, existen muchos preparados de melatonina con dosis muy similares a las administradas en el estudio comercializados como complemento alimenticio.

Se comercializan como complemento alimenticio preparados de melatonina de 1.9 mg. En este estudio la dosis a administrar es de 2 mg.

CONSIDERACIONES PRÁCTICAS

Responsabilidades de los participantes en el estudio

Normas para los sujetos participantes

El sujeto participante deberá seguir las indicaciones de los investigadores y comunicar cualquier eventualidad a los mismos.

El sujeto será debidamente informado de las prohibiciones o restricciones a las que deberá atenerse durante la realización del ensayo. El incumplimiento de estas recomendaciones implicará el abandono del estudio.

Todos los sujetos participantes en el estudio tienen derecho a abandonar el estudio en cualquier momento, retirando su consentimiento, sin tener que justificar esta decisión y sin que ello le suponga detrimento alguno en su seguimiento clínico. Si esto ocurre, el investigador intentará que el sujeto realice todas las evaluaciones necesarias para asegurar que no se presentan acontecimientos adversos y para garantizar un seguimiento apropiado en el caso de que se haya presentado algún tipo de problema.

Normas para el personal investigador

El investigador se compromete a cumplir con las normas establecidas en la legislación vigente en materia de ensayos clínicos.

Cumplimiento del protocolo

Se deberán evitar las desviaciones del protocolo. Si éstas ocurren, el investigador deberá informar al monitor y las implicaciones de dichas desviaciones serán revisadas y discutidas entre el equipo investigador. Las desviaciones del protocolo serán documentadas especificando los motivos, fecha, acción tomada, y el impacto en el paciente y en el ensayo. La documentación relativa a las desviaciones del protocolo será guardada en el Archivo del investigador.

Monitorización, auditoría e inspección

El estudio será monitorizado por Personal de la Plataforma Española de Ensayos Clínicos (SCReN) quien elaborará un plan de monitorización adecuado para el estudio. Se realizarán visitas regulares y llamadas telefónicas a los investigadores. Durante las visitas, el monitor deberá revisar los registros originales de los participantes, los registros de existencia de medicación y la conservación de los documentos. Además, el monitor deberá evaluar los procedimientos del estudio y discutir los eventuales problemas con el investigador. Durante el curso del estudio, se podrán llevar a cabo visitas de auditoría en los centros participantes. El investigador permitirá el acceso directo a los datos/documentos fuente para las tareas de monitorización, auditoría, revisión por el CEIm e inspección por las Autoridades Sanitarias.

Documentación del estudio

La documentación relativa al estudio (protocolo, CRD, CI, IB, autorizaciones, …) será archivada en la Unidad de Ensayos Clínicos del Hospital Universitario La Paz durante el estudio en un lugar seguro y fácilmente accesible por el equipo investigador. Toda la información contenida en informes clínicos, histológicos, bioquímicos y moleculares, observaciones u otras actividades es necesaria para la reconstrucción y evaluación del estudio. El monitor archivará estos documentos originales/ fuentes. Ejemplos de estos documentos son historial hospitalario, notas de laboratorio, memorándums, diario del paciente, listas de comprobaciones de evaluación, registro de dispensación del medicamento u otros documentos de farmacia, registro de los datos obtenidos de aparatos automatizados, archivos digitalizados de las fotografías, radiografías, etc.

Manejo y archivo de datos

Se diseñará y creará un cuaderno de recogida de datos electrónico (CRDe). Se diseñará y creará un Formulario Inicial MeCOVID y Formulario diario MeCOVID al que los participantes tendrán acceso a través de un enlace que recibirán por correo electrónico, diseñado para este estudio.

Únicamente los investigadores conocerán los datos (iniciales del paciente y número de historia) que puedan identificar a los participantes. Las iniciales y el número de historia del paciente se separarán del resto del cuaderno, y no serán introducidos en la base de

datos del estudio. El paciente será identificado mediante un código numérico con la finalidad de respetar la confidencialidad de los datos personales de los participantes, según establece el Reglamento (UE) 2016/679 del Parlamento europeo y del Consejo de 27 de abril de 2016 de Protección de Datos (RGPD). La colección y el manejo de muestras seguirá las indicaciones establecidas en la Ley 14/2007, de 3 de julio, de investigación biomédica.

Es responsabilidad de los investigadores la preparación de un manuscrito en un plazo máximo de 6 meses a contar desde que se cierre la base de datos del estudio para su publicación en una revista de la especialidad. Los datos globales serán utilizados en publicaciones en congresos o revistas científicas/médicas, en las que se hará referencia al estudio y al grupo investigador.

En el momento de su registro se asignará a cada paciente un Número de Paciente. Este Número de Paciente y las iniciales del paciente (primera letra del nombre, primer apellido y segundo apellido) se anotarán en el CRD.

El investigador será responsable de conservar la información adecuada acerca de cada paciente de forma que las autoridades sanitarias puedan tener acceso a dicha información si así fuera preciso. Estos registros deberán conservarse de manera confidencial durante el período de tiempo legalmente ordenado por la normativa vigente.

Condiciones de publicación

Toda la información procedente del estudio será considerada confidencial. El Investigador Principal asume el conjunto de responsabilidades ligado a esta función, y a la propiedad exclusiva de los resultados del estudio, que podrá explotar libremente, comprometiéndose a publicar los resultados del estudio en una revista científica o a ponerlos a disposición del público tal y como establece la Declaración de Helsinki en el punto 27: “Tanto los autores como los editores tienen obligaciones éticas. Al publicar los resultados de su investigación, el médico está obligado a mantener la exactitud de los datos y resultados. Se deben publicar tanto los resultados negativos como los positivos o de lo contrario deben estar a la disposición del público. En la publicación se debe citar la fuente de financiamiento, afiliaciones institucionales y cualquier conflicto de intereses. Los informes sobre investigaciones que no se ciñan a los principios descritos en esta Declaración no deben ser aceptados para su publicación"

Procedimiento para las modificaciones del protocolo

Cualquier modificación al protocolo debe documentarse por escrito en forma de enmienda. Las enmiendas estarán debidamente identificadas por su número de orden cronológico, fechadas y firmadas por el promotor y el investigador. Si las

modificaciones son relevantes, el Promotor deberá solicitar autorización al CEIC de Referencia y a la AMPES, según establece la normativa vigente.

Comité ético de investigación con medicamentos (CEIm)

El protocolo y los documentos de consentimiento informado serán evaluados por el CEIm del Hospital Universitario La Paz cuya composición es ya conocida y acreditada por las autoridades sanitarias de la Comunidad Autónoma de Madrid (http://www.msssi.gob.es/profesionales/farmacia/ceic/pdf/ceicsacreditadoses.pdf). La decisión del CEIC referente al desarrollo del estudio se le facilitará por escrito al investigador; una copia de dicha decisión deberá ser remitida al promotor.

El promotor presentará los informes requeridos de progreso del estudio al CEIm, y comunicará las sospechas de reacción adversa graves e inesperadas. A la terminación del estudio, el promotor deberá informar de ello al CEIm.

ANALISIS ESTADÍSTICO

Se realizará un análisis descriptivo-univariante de todas las variables clínicas y analíticas estudiadas. Éstas se presentarán en frecuencias absolutas y relativas en caso de variables cualitativas y las principales medidas de centralización y dispersión (media, desv. típica, mediana, mínimo, máximo, rango intercuartílico (RI) e intervalo de confianza al 95% (IC95%)), en caso de variables cuantitativas. Se estudiará la normalidad de las variables mediante la prueba de normalidad de Kolmogorov-Smirnof.

Para comparaciones de muestras independientes, se utilizará el test Chi-Cuadrado de Pearson (o la prueba exacta de Fisher para tablas 2X2 o razón de verosimilitud en tablas mXn, en caso necesario) si se trata de variables cualitativas y el test T_Student, ANOVA de un factor o sus equivalentes no paramétricos U de_Mann-Whitney, prueba de Kruskal-Wallis en caso de variables cuantitativas.

Antes del inicio del ensayo clínico se elaborará un borrador del Plan de Análisis Estadístico (PAE). El PAE y el análisis se llevará a cabo por el personal de la Unidad Central de Investigación Clínica y Ensayos Clínicos del Hospital Universitario La Paz (UCICEC).

BLBIBLIOGRAFÍA

1. INFORME TÉCNICO. Enfermedad por coronavirus, COVID-19. Actualización; 17 de marzo 2020. Ministerio de Sanidad. Disponible en: https://www.mscbs.gob.es/profesionales/saludPublica/ccayes/alertasActual/nCov-China/documentos/20200317_ITCoronavirus.pdf
2. Novel Coronavirus (2019-nCoV) situation reports [Internet]. [citado 19 de Marzo de 2020]. Disponible en: https://www.who.int/docs/default-source/coronaviruse/situation-reports/20200320-sitrep-60-covid-19.pdf?sfvrsn=8894045a_2
3. Zhou D1,2, Dai SM3, Tong Q3. COVID-19: a recommendation to examine the effect of hydroxychloroquine in preventing infection and progression. J Antimicrob Chemother. 2020 Mar 20. pii: dkaa114. doi: 10.1093/jac/dkaa114. [Epub ahead of print]
4. Wang D, Hu B, Hu C et al. Clinical characteristics of 138 hospitalized patients with 2019 novel coronavirus-infected pneumonia in Wuhan, China. AMA 2020; doi:10.1001/jama.2020.1585.
5. Lee PI, Hu YL, Chen PY, Huang YC, Hsueh PR. Are children less susceptible to COVID-19? Journal of microbiology, immunology, and infection = Wei mian yu gan ran za zhi. 2020.
6. Wu Z, McGoogan JM. Characteristics of and Important Lessons From the Coronavirus Disease 2019 (COVID-19) Outbreak in China: Summary of a Report of 72 314 Cases From the Chinese Center for Disease Control and Prevention. Jama. 2020.
7. Lu X, Zhang L, Du H, Zhang J, Li YY, Qu J, et al. SARS-CoV-2 Infection in Children. The New England journal of medicine. 2020.
8. Zhou F, Yu T, Du R, Fan G, Liu Y, Liu Z, et al. Clinical course and risk factors for mortality of adult inpatients with COVID-19 in Wuhan, China: a retrospective cohort study. Lancet (London, England). 2020.
9. Zhang J-j, Dong X, Cao Y-y, Yuan Y-d, Yang Y-b, Yan Y-q, et al. Clinical characteristics of 140 patients infected with SARS-CoV-2 in Wuhan, China. Allergy.n/a.
10. Scholtens RM, van Munster BC, van Kempen MF, de Rooij SE. Physiological melatonin levels in healthy older people: A systematic review. Journal of psychosomatic research. 2016;86:20-7.
11. Grivas TB, Savvidou OD. Melatonin the "light of night" in human biology and adolescent idiopathic scoliosis. Scoliosis. 2007;2:6.
12. Gunn PJ, Middleton B, Davies SK, Revell VL, Skene DJ. Sex differences in the circadian profiles of melatonin and cortisol in plasma and urine matrices under constant routine conditions. Chronobiology international. 2016;33:39-50.
13. Cipolla-Neto J, Amaral FG, Afeche SC, Tan DX, Reiter RJ. Melatonin, energy metabolism, and obesity: a review. Journal of pineal research. 2014;56:371-81.
14. Sun H, Gusdon AM, Qu S. Effects of melatonin on cardiovascular diseases: progress in the past year. Current opinion in lipidology. 2016;27:408-13.

15. Pechanova O, Paulis L, Simko F. Peripheral and central effects of melatonin on blood pressure regulation. International journal of molecular sciences. 2014;15:17920-37.
16. Zhang R, Wang X, Ni L, Di X, Ma B, Niu S, et al. COVID-19: Melatonin as a potential adjuvant treatment. Life Sciences. 2020:117583.
17. Ruan Q, Yang K, Wang W, Jiang L, Song J. Clinical predictors of mortality due to COVID-19 based on an analysis of data of 150 patients from Wuhan, China. Intensive Care Medicine. 2020.
18. Shiu SY, Reiter RJ, Tan DX, Pang SF. Urgent search for safe and effective treatments of severe acute respiratory syndrome: is melatonin a promising candidate drug? Journal of pineal research. 2003;35:69-70.
19. Mohan N, Sadeghi K, Reiter RJ, Meltz ML. The neurohormone melatonin inhibits cytokine, mitogen and ionizing radiation induced NF-kappa B. Biochemistry and molecular biology international. 1995;37:1063-70.
20. Poeggeler B, Reiter RJ, Tan DX, Chen LD, Manchester LC. Melatonin, hydroxyl radical-mediated oxidative damage, and aging: a hypothesis. Journal of pineal research. 1993;14:151-68.
21. Reiter RJ. Oxygen radical detoxification processes during aging: the functional importance of melatonin. Aging (Milan, Italy). 1995;7:340-51.
22. Tan DX, Manchester LC, Reiter RJ, Qi WB, Karbownik M, Calvo JR. Significance of melatonin in antioxidative defense system: reactions and products. Biological signals and receptors. 2000;9:137-59.
23. Cuzzocrea S, Reiter RJ. Pharmacological actions of melatonin in acute and chronic inflammation. Current topics in medicinal chemistry. 2002;2:153-65.
24. Bonilla E, Rodon C, Valero N, Pons H, Chacin-Bonilla L, Garcia Tamayo J, et al. Melatonin prolongs survival of immunodepressed mice infected with the Venezuelan equine encephalomyelitis virus. Transactions of the Royal Society of Tropical Medicine and Hygiene. 2001;95:207-10.
25. Bonilla E, Valero N, Chacin-Bonilla L, Medina-Leendertz S. Melatonin and viral infections. Journal of pineal research. 2004;36:73-9.
26. Bonilla E, Valero-Fuenmayor N, Pons H, Chacin-Bonilla L. Melatonin protects mice infected with Venezuelan equine encephalomyelitis virus. Cellular and molecular life sciences : CMLS. 1997;53:430-4.
27. Gitto E, Karbownik M, Reiter RJ, Tan DX, Cuzzocrea S, Chiurazzi P, et al. Effects of melatonin treatment in septic newborns. Pediatric research. 2001;50:756-60.
28. Reiter RJ, Tan DX, Sainz RM, Mayo JC, Lopez-Burillo S. Melatonin: reducing the toxicity and increasing the efficacy of drugs. The Journal of pharmacy and pharmacology. 2002;54:1299-321.
29. Anderson G, Maes M, Markus RP, Rodriguez M. Ebola virus: melatonin as a readily available treatment option. Journal of medical virology. 2015;87:537-43.
30. Masters A, Pandi-Perumal SR, Seixas A, Girardin JL, McFarlane SI. Melatonin, the Hormone of Darkness: From Sleep Promotion to Ebola Treatment. Brain disorders & therapy. 2014;4.

31. Tan DX, Korkmaz A, Reiter RJ, Manchester LC. Ebola virus disease: potential use of melatonin as a treatment. Journal of pineal research. 2014;57:381-4.
32. Wirtz PH, Spillmann M, Bärtschi C, Ehlert U, von Känel R. Oral melatonin reduces blood coagulation activity: a placebo-controlled study in healthy young men. Journal of pineal research. 2008;44:127-33.
33. Nunes Oda S, Pereira Rde S. Regression of herpes viral infection symptoms using melatonin and SB-73: comparison with Acyclovir. Journal of pineal research. 2008;44:373-8.
34. Tan DX, Zheng X, Kong J, Manchester LC, Hardeland R, Kim SJ, et al. Fundamental issues related to the origin of melatonin and melatonin isomers during evolution: relation to their biological functions. International journal of molecular sciences. 2014;15:15858-90.
35. Nunn AV, Guy GW, Bell JD. The quantum mitochondrion and optimal health. Biochemical Society transactions. 2016;44:1101-10.
36. Singer M. The role of mitochondrial dysfunction in sepsis-induced multi-organ failure. Virulence. 2014;5:66-72.
37. Choi D. Potency of melatonin in living beings. Development & reproduction. 2013;17:149-77.
38. Reiter RJ, Mayo JC, Tan D-X, Sainz RM, Alatorre-Jimenez M, Qin L. Melatonin as an antioxidant: under promises but over delivers. Journal of pineal research. 2016;61:253-78.
39. Acuña-Castroviejo D, Carretero M, Doerrier C, López LC, García-Corzo L, Tresguerres JA, et al. Melatonin protects lung mitochondria from aging. Age (Dordrecht, Netherlands). 2012;34:681-92.
40. Kelley N, Jeltema D, Duan Y, He Y. The NLRP3 Inflammasome: An Overview of Mechanisms of Activation and Regulation. International journal of molecular sciences. 2019;20.
41. Favero G, Franceschetti L, Bonomini F, Rodella LF, Rezzani R. Melatonin as an Anti-Inflammatory Agent Modulating Inflammasome Activation. International journal of endocrinology. 2017;2017:1835195.
42. Bazyar H, Gholinezhad H, Moradi L, Salehi P, Abadi F, Ravanbakhsh M, et al. The effects of melatonin supplementation in adjunct with non-surgical periodontal therapy on periodontal status, serum melatonin and inflammatory markers in type 2 diabetes mellitus patients with chronic periodontitis: a double-blind, placebo-controlled trial. Inflammopharmacology. 2019;27:67-76.
43. Sánchez-López AL, Ortiz GG, Pacheco-Moises FP, Mireles-Ramírez MA, Bitzer-Quintero OK, Delgado-Lara DLC, et al. Efficacy of Melatonin on Serum Pro-inflammatory Cytokines and Oxidative Stress Markers in Relapsing Remitting Multiple Sclerosis. Archives of Medical Research. 2018;49:391-8.
44. Boga JA, Coto-Montes A, Rosales-Corral SA, Tan DX, Reiter RJ. Beneficial actions of melatonin in the management of viral infections: a new use for this "molecular handyman"? Reviews in medical virology. 2012;22:323-38.

45. Srinivasan V, Mohamed M, Kato H. Melatonin in bacterial and viral infections with focus on sepsis: a review. Recent patents on endocrine, metabolic & immune drug discovery. 2012;6:30-9.
46. Zhou Y, Hou Y, Shen J, Huang Y, Martin W, Cheng F. Network-based drug repurposing for novel coronavirus 2019-nCoV/SARS-CoV-2. Cell discovery. 2020;6:14.
47. Zhang H, Penninger JM, Li Y, Zhong N, Slutsky AS. Angiotensin-converting enzyme 2 (ACE2) as a SARS-CoV-2 receptor: molecular mechanisms and potential therapeutic target. Intensive Care Med. 2020;46:586-90.
48. Bubenik GA, Konturek SJ. Melatonin and aging: prospects for human treatment. J Physiol Pharmacol. 2011 Feb;62(1):13-9. PMID: 21451205.
49. Vural EM, van Munster BC, de Rooij SE. Optimal dosages for melatonin supplementation therapy in older adults: a systematic review of current literature. Drugs Aging. 2014 Jun;31(6):441-51. doi: 10.1007/s40266-014-0178-0. PMID: 24802882.
50. Zhdanova IV, Wurtman RJ, Balcioglu A, Kartashov AI, Lynch HJ. Endogenous melatonin levels and the fate of exogenous melatonin: age effects. J Gerontol A Biol Sci Med Sci. 1998 Jul;53(4):B293-8. PMID: 18314560
51. Gooneratne NS, Edwards AY, Zhou C, Cuellar N, Grandner MA, Barrett JS. Melatonin pharmacokinetics following two different oral surge-sustained release doses in older adults. J Pineal Res. 2012 May;52(4):437-45. doi: 10.1111/j.1600-079X.2011.00958.x. Epub 2012 Feb 21. PMID: 22348451.
52. Ran L, Chen X, Wang Y, Wu W, Zhang L, Tan X.Risk Factors of Healthcare Workers with Corona Virus Disease 2019: A Retrospective Cohort Study in a Designated Hospital of Wuhan in China. Clin Infect Dis. 2020 Mar 17. pii: ciaa287. doi: 10.1093/cid/ciaa287. [Epub ahead of print]
53. Guía de actuación frente a COVID-19 en los profesionales sanitarios y sociosanitarios Versión del 31 de marzo de 2020. Disponible en: https://www.mscbs.gob.es/profesionales/saludPublica/ccayes/alertasActual/nCov-China/documentos/Protocolo_Personal_sanitario_COVID-19.pdf

54. National Early Warning Score (NEWS) 2. Disponible en: https://www.rcplondon.ac.uk/projects/outputs/national-early-warning-score-news-2

ANEXO 1. The NEWS scoring system[54]

The NEWS is based on a simple aggregate scoring system in which a score is allocated to physiological measurements, already recorded in routine practice, when patients present to, or are being monitored in hospital. Six simple physiological parameters form the basis of the scoring system:

1. respiration rate
2. oxygen saturation
3. systolic blood pressure
4. pulse rate
5. level of consciousness or new confusion*
6. temperature.

**The patient has new-onset confusion, disorientation and/or agitation, where previously their mental state was normal – this may be subtle. The patient may respond to questions coherently, but there is some confusion, disorientation and/or agitation. This would score 3 or 4 on the GCS (rather than the normal 5 for verbal response), and scores 3 on the NEWS system.*

A score is allocated to each parameter as they are measured, with the magnitude of the score reflecting how extremely the parameter varies from the norm. The score is then aggregated and uplifted by 2 points for people requiring supplemental oxygen to maintain their recommended oxygen saturation.

Physiological parameter	Score						
	3	2	1	0	1	2	3
Respiration rate (per minute)	≤8		9–11	12–20		21–24	≥25
SpO_2 Scale 1 (%)	≤91	92–93	94–95	≥96			
SpO_2 Scale 2 (%)	≤83	84–85	86–87	88–92<br>≥93 on air	93–94 on oxygen	95–96 on oxygen	≥97 on oxygen
Air or oxygen?		Oxygen		Air			
Systolic blood pressure (mmHg)	≤90	91–100	101–110	111–219			≥220
Pulse (per minute)	≤40		41–50	51–90	91–110	111–130	≥131
Consciousness				Alert			CVPU
Temperature (°C)	≤35.0		35.1–36.0	36.1–38.0	38.1–39.0	≥39.1	

ANEXO 2: Escala ordinal de 7-puntos para evaluación del status clínico

1. ALTA (o altable: Tª normal, satO2 estable en aire ambiente o requerimientos de O2 menor o igual a 2L por minuto).
2. INGRESADO: No UCI o No requiere O2
3. INGRESADO NO UCI, pero requiere O2 con gafas nasales o ventimask
4. INGRESADO UCI o NO, pero requiere O2 con reservorio
5. INGRESADO UCI o NO, requiriendo ventilación mecánica no invasiva o gafas de alto flujo
6. INGRESADO UCI, con ventilación mecánica invasiva
7. MUERTE
